# Healthcare professionals’ knowledge, attitudes, and practices regarding graduated compression stockings: a survey of China’s big-data network

**DOI:** 10.1186/s12913-020-05933-9

**Published:** 2020-11-25

**Authors:** Yaping Xu, Wei Wang, Kaiyuan Zhen, Jing Zhao

**Affiliations:** 1 Department of Orthopedics, China-Japan Friendship Hospital, 2 Yinghua Dongjie, Hepingli, Chaoyang District, Beijing, 100029 China; 2 Bone Necrosis and Joint Preservation Reconstruction Center, China-Japan Friendship Hospital, Beijing, China; 3 Department of Nursing, China-Japan Friendship Hospital, Beijing, China; 4 Institute of Clinical Medical Sciences, China-Japan Friendship Hospital, Beijing, China

**Keywords:** Graduated compression stockings, Venous thromboembolism, Knowledge, Attitude, Practice

## Abstract

**Background:**

The accurate identification of venous thromboembolism prophylaxis implementation barriers is an important part of prophylaxis prevention. However, in China, data to help identify these barriers is limited. This study has two objectives: 1) to determine the knowledge, attitudes, and practices (KAPs) of healthcare professionals regarding graduated compression stockings (GCS) since the launch of the National Program for the Prevention and Management of Pulmonary Embolism (PE) and Deep Venous Thrombosis (DVT) in October 2018 and 2) to identify the obstacles and assist the program.

**Methods:**

This was a cross-sectional study of 5070 healthcare professionals in China. We used exploratory factor and reliability analyses to evaluate the researcher-designed questionnaire’s reliability and validity. The formal questionnaire, which included demographic data, knowledge, attitudes, and clinical practice patterns, was distributed to healthcare professionals.

**Results:**

Of the 5070 respondents, 32.5% had a good knowledge of GCS, 78.5% had a positive attitude towards their use, and 34.0% exhibited normative behavior when applying them. The KAPs of healthcare professionals towards GCS were significantly correlated with one another. Binary logistic regression suggested that the training received by healthcare professionals was an important factor affecting their knowledge regarding GCS usage.

**Conclusions:**

The training provided for the use of GCS in China cannot meet medical staff needs and deserves more attention from policy makers. This represents an obstacle for venous thromboembolism prophylaxis, which restricts the effective implementation of the National Program for Prevention and Management of PE and DVT.

## Background

Venous thromboembolism (VTE) is the third-leading cause of cardiovascular-associated deaths worldwide [1]. Effective VTE prevention is an intense research area, and many guidelines [2–8] have been developed regarding VTE prophylaxis. Some studies suggest that the existence of a large real-world gap between Western evidence-based guidelines and the current knowledge of healthcare professionals represents an impediment to effective VTE prophylaxis implementation [9–13]. Accurately identifying the barriers to VTE prophylaxis implementation is an important part of VTE prevention. However, in China, data to help identify these VTE prophylaxis barriers is limited.

The National Program for the Prevention and Management of Pulmonary Embolism (PE) and Deep Venous Thrombosis (DVT) was launched in October 2018 in China. This program aims to improve the overall ability of hospitals to prevent, diagnose, and treat VTE. In this study, we aimed to determine the knowledge, attitude, and practices (KAPs) conducted by healthcare professionals regarding graduated compression stockings (GCS) since the launch of the National Program to identify obstacles and assist this program. To the best of our knowledge, our study is the first to explore the KAPs regarding the use of GCS by healthcare professionals accredited by the National Program and provide a basis for follow-up work.

## Methods

### Study design

This was a non-interventional, anonymized, self-administered, one-time web-based survey for healthcare professionals in China. Based on the National Program for Prevention and Management of PE and DVT, this survey was conducted from November 19 to December 5, 2019.

### Survey questionnaire

The questionnaire items were designed by experts from the working group for the National Program for Prevention and Management of PE and DVT. An original questionnaire that included 31 items was developed based on a preliminary survey of 928 cases to evaluate its reliability and validity. In the pre-investigation, researchers screened all items and created a formal questionnaire through exploratory factor analysis (EFA) using IBM SPSS Statistics for Windows version 25.0 (IBM Corp., Armonk, NY, USA). The questionnaire, which had a high Cronbach’s alpha coefficient (0.952), was designed by experts from the National Program (Supplementary File 1). The 31-item validated questionnaire was used to collect demographic data (9 items), knowledge (11 items), attitude (4 items), and clinical practice patterns (7 items). The Cronbach’s alpha for knowledge, attitude, and clinical practice pattern scales were 0.951, 0.910, and 0.961, respectively.

Demographical data included the following characteristics: gender, age, highest level of education attained, profession, hospital level, service years, professional title, administrative duties, and GCS usage training. The questionnaire did not ask for any personal identifying information. The other 22 items were single-choice questions, which were scored on a 5-point Likert scale ranging from 1 to 5. The higher the score, the better the result.

The 11 items in the knowledge dimension were assessed using a 5-point Likert scale that was as follows: 1 = very unfamiliar, 2 = unfamiliar, 3 = generally familiar, 4 = familiar, and 5 = very familiar. Higher scores represented better knowledge. Further, the 4 items in the attitude dimension were evaluated as follows: 1 = strongly disagree, 2 = disagree, 3 = generally agree, 4 = agree, and 5 = strongly agree. Higher scores indicated a more positive attitude. Finally, the 7 items in the clinical practice dimension were scored as follows: 1 = never, 2 = seldom, 3 = occasionally, 4 = often, and 5 = frequently. Higher scores represented a more normative behavior. Hence, a score of 4 and 5 points in any of the dimensions was considered good.

### Data collection

This was a closed survey. The researchers uploaded the questionnaire to the Sojump online platform (https://www.wjx.cn/), which generated a QR code. The QR code was then distributed to the certifying agency’s liaisons through the National Program’s platform. The participants used the QR code through the WeChat application (Tencent Holdings, Shenzhen, China), and responded to and submitted the questionnaire.

The questionnaire was designed in such a way that it could not be submitted until all questions are answered. Participants could review and change their responses before submission. The survey period lasted for 18 days. During this time, 5070 healthcare professionals responded to the survey.

### Statistical analysis

All data analyses were performed using IBM SPSS Statistics for Windows version 25.0 (IBM Corp., Armonk, NY, USA). Exploratory factor analysis and reliability analysis were used to evaluate the reliability and validity of this researcher-designed questionnaire. Descriptive statistical analysis was used to summarize the demographic characteristics of the 5070 healthcare professionals. Continuous data were expressed as means and standard deviations and were compared using Student’s *t*-test. Categorical data were reported as absolute numbers and proportions and were compared using the Chi-Squared test. Pearson’s correlation coefficients were computed to examine the relationship between the KAPs and GCS clinical usage. Binary logistic regression was used to analyze the factors that influenced the classification of “good” and “not good” for the knowledge dimension of healthcare professionals towards GCS clinical usage, and 95% confidence intervals were calculated. A two-tailed *p*-value higher than 0.05 was considered statistically significant.

## Results

### Demographic characteristics

Demographic characteristics are shown in Table 1. Female medical staff represented the majority of respondents (91.0%). Further, a major proportion of the respondents had a bachelor’s degree or a higher academic degree (80.5%). Moreover, many of the respondents were nurses (87.1%), most of whom worked in tertiary care hospitals (89.2%), and 63.4% of respondents received GCS usage training.

**Table 1:** Respondent characteristics (*N* = 5070)

Characteristic	Categories	*n*	%
Gender	Men	454	9.0
	Women	4616	91.0
Age (years)	< 29	1999	39.4
	30–39	2180	43.0
	≥ 40	891	17.6
Highest education attained	Secondary ^a^	67	1.3
	College	921	18.2
	Bachelor’s degree	3659	72.2
	Master’s degree	382	7.5
	Doctoral degree	41	0.8
Profession	Doctor	654	12.9
	Nurse	4416	87.1
Hospital level	Tertiary hospital	4523	89.2
	Secondary hospital	547	10.8
Service years	1–5	1495	29.5
	6–10	1501	29.6
	11–19	1271	25.1
	≥ 20	803	15.8
Professional title	Junior	2992	59.0
	Intermediate	1688	33.3
	Senior	390	7.7
Administrative duties	No	4422	87.2
	Education secretary	337	6.7
	Head nurse	205	4.0
	Doctor director	106	2.1
GCS application training	Yes	3216	63.4
	No ^b^	1854	36.6

*GCS* Graduated compression stockings

^a^Even in some tertiary hospitals in China, there are a few nurses who have worked for more than 30 years who have only completed secondary education

^b^Ideally, all healthcare professionals in hospitals accredited by the National Program for Prevention and Management of primary embolism and deep vein thrombosis should have training for the use of GCS; however, the survey shows a wide gap in the implementation of the program in the real world

### KAPs of healthcare professionals regarding GCS usage

**Fig. 1:**
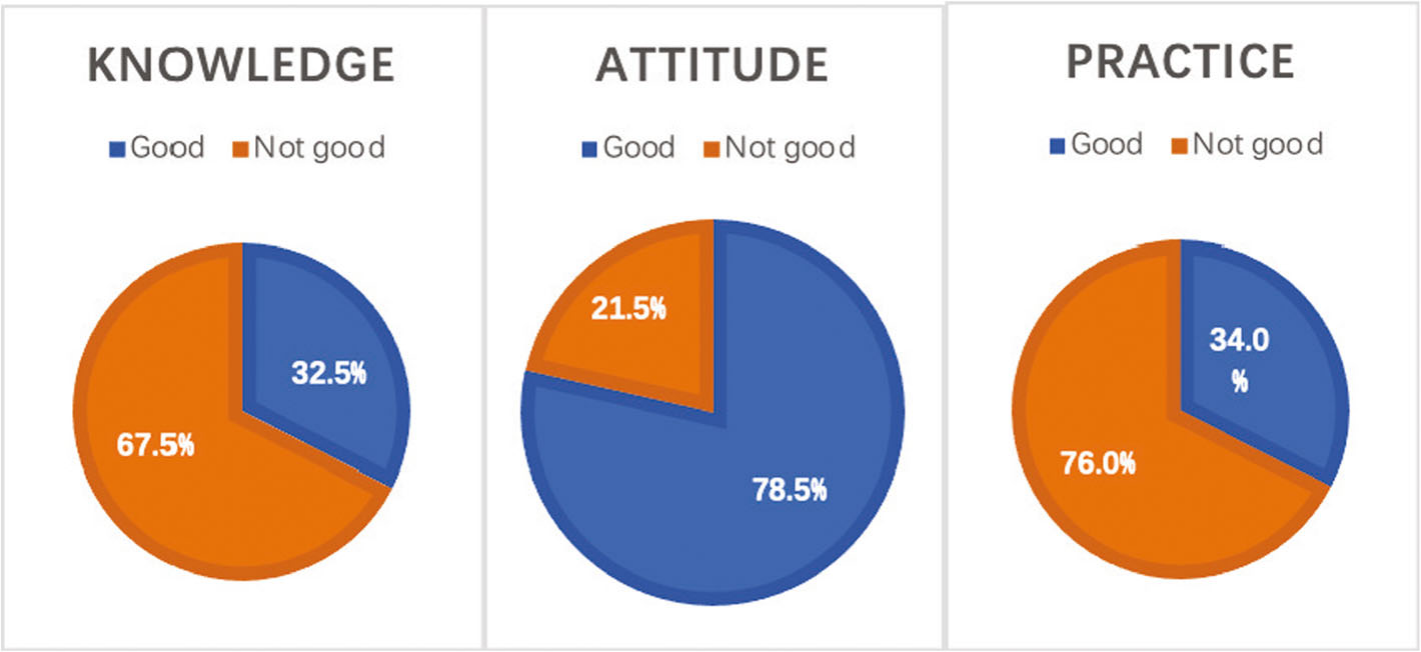
Proportion of “good” classifications for KAP dimensions (*N* = 5070)

**Fig. 2:**
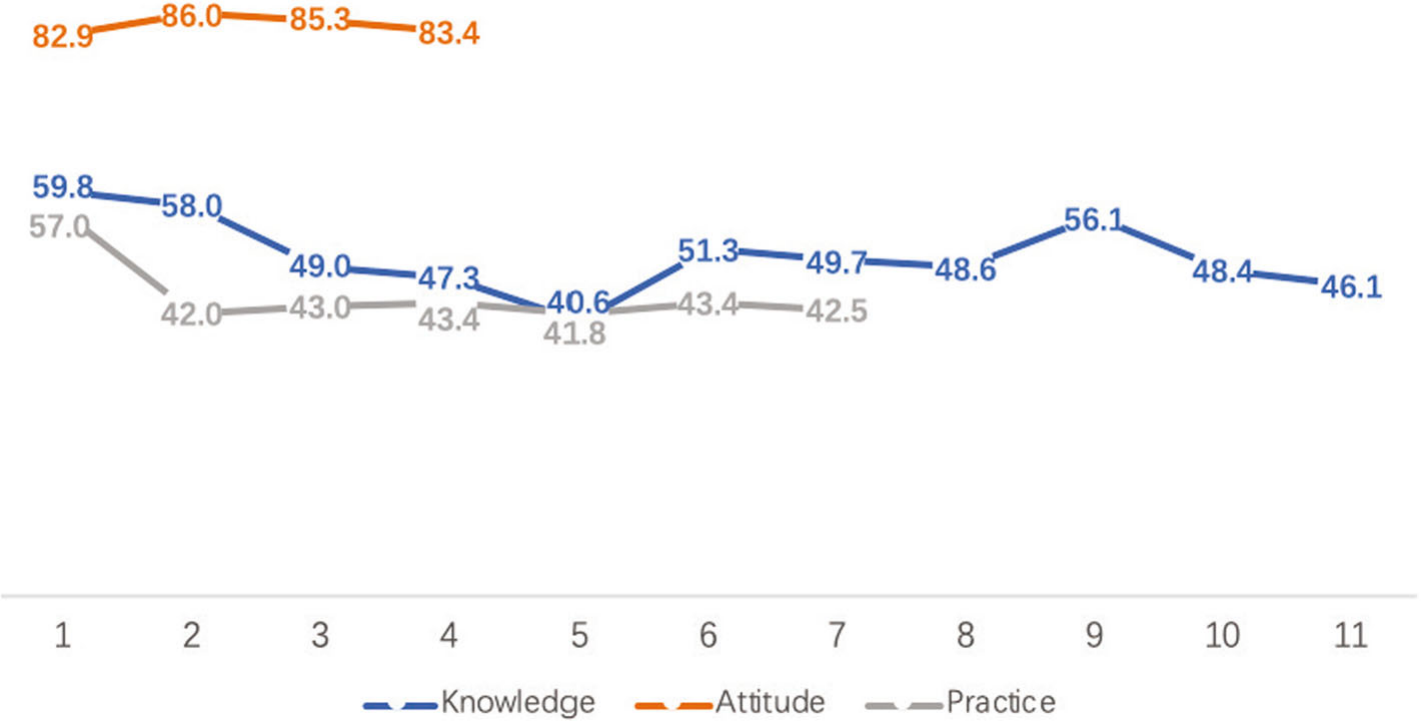
Proportion of “good” classifications for the 22 KAP dimension items (*N* = 5070). *Knowledge (11 items) 1.* Mechanism of action; *2.* Indications; *.3.* Contraindications; *4.* Size; *5.* Pressure level; *6.* Length; *7.* Timing; *8.* Wearing method; *9.* Maintenance instructions; *10.* Washing method; and *11.* Service life. *Attitude (4 items) 1. GCS benefits* should be actively communicated to patients and their caregivers; *2.* Medical staff should teach patients and their caregivers the proper use of GCS; *3.* Medical institutions and managers should pay attention to the training of healthcare professionals for GCS usage; and *4.* GCS should be covered by Medicare. *Practice (7 items) 1.* I think my guidance on GCS usage for patients is in place; *2.* I make sure that the patients in my charge have been well informed of GCS usage benefits; *3.* I make sure that the patients in my charge are well aware of the importance of the proper use of GCS upon discharge; *4.* I make sure that the patients in my charge are capable of wearing GCS independently or with the help of the caregivers when they leave the hospital; *5.* I make sure that the patients under my charge know how to solve problems (such as skin indentation, blisters or discoloration) associated with GCS usage, especially at the ankle or protuberance, after discharge; *6.* I make sure that at discharge I have made clear to the patients in my charge who to contact in case of GCS misusage; and *7.* I make sure that at discharge I have made it clear to the patients in my charge when to stop using GCS

Figure 1 shows that of the 5070 respondents, 32.5% had good knowledge regarding GCS usage; 78.5% had a positive attitude towards using GCS, and 34.0% exhibited normative behavior when using GCS. The proportions of “good” classifications for all the 22 KAP dimension items are illustrated in Fig. 2.

Table 2 indicates that the KAPs of healthcare professionals regarding clinical GCS usage significantly correlated with one another. There was a significantly positive, moderate correlation between knowledge and attitude (*r* = 0.463, *p* < 0.01). Furthermore, there was a significantly positive strong correlation between knowledge and practice (*r* = 0.658, *p* < 0.01), and a significantly positive moderate correlation between attitude and practice (*r* = 0.428, *p* < 0.01).

**Table 2:** KAP means, standard deviations, and Pearson’ s correlation coefficients (*N* = 5070)

Variables	*M*	*SD*	Minimum	Maximum	Knowledge	Attitude	Behavior
Knowledge	38.18	9.098	11	55	1		
Attitude	16.65	2.656	4	20	0.463*	1	
Behavior	22.22	8.227	7	35	0.658*	0.428*	1

The correlation is significant at 0.01 (two-tailed)

*M* Mean, *SD* Standard deviation

*p < 0.01

### Factors from demographic characteristics that influenced the “good” and “not good” classifications in the knowledge dimension

A single-factor analysis suggested that the independent variables that influenced the “good” and “not good” classifications in the knowledge dimension were statistically significant (*p* < 0.05; *p* < 0.01) and included gender, age, hospital level, service years, administrative duties, and GCS usage training (Table 3). These variables were incorporated into a binary logistic regression model. Compared with men, women were less likely to have good knowledge regarding GCS clinical usage (OR = 0.772, 95% CI 0.605–0.986) (Table 4). Moreover, compared with those who received GCS usage training, those who did not receive training had a very low probability of having good knowledge regarding GCS clinical usage (OR = 0.097, 95% CI 0.081–0.117).

**Table 3:** Single-factor comparison of the demographic characteristics that influence “good” and “not good” classifications in the knowledge dimension

	Good (*n* = 1647)	Not good (*n* = 3423)	*χ*^*2*^	*P*
Gender, Men (%)	167 (10.1)	287 (8.4)	4.202^a^	0.040*
Age (years)			26.331^a^	0.000**
<29	733 (44.5)	1266 (37.0)		
30–39	649 (39.4)	1531 (44.7)		
≥ 40	265 (16.1)	626 (18.3)		
Highest education attained			5.955^a^	0.203
Secondary	20 (1.2)	47 (1.4)		
College	279 (16.9)	642 (18.8)		
Bachelor’s degree	1217 (73.9)	2442 (71.3)		
Master’s degree	114 (6.9)	268 (7.8)		
Doctoral degree	17 (1.0)	24 (0.7)		
Profession			0.159^a^	0.690
Doctor	208 (12.6)	446 (13.0)		
Nurse	1439 (87.4)	2977 (87.0)		
Hospital level			4.649^a^	0.031*
Tertiary hospital	1447 (87.9)	3076 (89.9)		
Secondary hospital	200 (12.1)	347 (10.1)		
Service years			16.063^a^	0.001**
1–5	542 (32.9)	953 (27.8)		
6–10	482 (29.3)	1019 (29.8)		
11–19	390 (23.7)	881 (25.7)		
≥ 20	233 (14.1)	570 (16.7)		
Professional title			2.001^a^	0.368
Junior	995 (60.4)	1997 (58.3)		
Intermediate	531 (32.2)	1157 (33.8)		
Senior	121 (7.3)	269 (7.9)		
Administrative duties			12.664^a^	0.005**
No	1420 (86.2)	3002 (87.7)		
Education				
Secretary	130 (7.9)	207 (6.0)		
Head nurse	54 (3.3)	151 (4.4)		
Doctor director	43 (2.6)	63 (1.8)		
GCS application training			810.688^a^	0.000**
Yes	1502 (91.2)	1714 (50.1)		
No	145 (8.8)	1709 (49.9)		

^a^Pearson Chi-Square test

*p < 0.05; **p < 0.01

**Table 4:** The factors that influence “good” and “not good” classifications in the knowledge dimension in binary logistic regression analysis

	95%CI (Lower bound/upper bound)	*P*
Gender		0.038
Men	1	*
Women	0.772 (0.605–0.986)	
Age (years)		0.110
<29	1	
30–39	0.794 (0.639–0.985)	
≥ 40	0.764 (0.487–1.198)	
Hospital level		0.684
Tertiary hospital	1	
Secondary hospital	0.958 (0.781–1.176)	
Service years		0.668
1–5	1	
6–10	0.983 (0.805–1.201)	
11–19	0.958 (0.730–1.256)	
≥ 20	0.748 (0.462–1.211)	
Administrative duties		0.050
No	1	
Education secretary	1.253 (0.962–1.633)	
Head nurse	0.806 (0.570–1.140)	
Doctor director	1.602 (0.992–2.586)	
GCS application training		0.000**
Yes	1	
No	0.097 (0.081–0.117)	

*CI* Confidence interval

*p < 0.05; **p < 0.01

## Discussion

### Current situation of the KAPs of healthcare professionals regarding GCS clinical usage

A majority of the respondents reported a positive attitude towards GCS clinical usage. The lowest proportion of “good” classifications for the 11 items in the knowledge dimension and the 7 items in the practice dimension were 40.6 and 41.8%, respectively, which was higher than that reported in previous surveys [11, 13]. These results depended on the implementation of the National Program. Considering that all the participants in this survey were from hospitals accredited by the National Program, we expected a higher proportion of “good” classifications for the knowledge and practice dimensions. A lower proportion may be suggestive of a substantial gap in the implementation of the program in the real world. Our survey indicates that the National Program should pay attention to the knowledge and practice dimensions in their future work. This result made the National Program members suspect that more detailed supervision and verification were necessary when the hospital was authorized. Simultaneously, providing uniform training courses to improve the work of the National Program is necessary. Regular reviews of authorized hospitals are necessary in the future.

### Improve the knowledge of healthcare professionals regarding GCS clinical usage

Our study shows that the KAPs of healthcare professionals regarding GCS clinical usage were significantly correlated with each other. Furthermore, although knowledge profoundness is particularly important, only 32.5% of the respondents had good knowledge regarding GCS usage, which was not encouraging. The National Program team recommends that the knowledge of healthcare professionals regarding GCS usage must include the following: action mechanism, indications, contraindications, sizes, pressure levels, lengths, usage timing, wearing methods, maintenance instructions, washing methods, and service life. These recommendations are consistent with the views of other scholars [14–16].

### Focus on GCS usage training

A single-factor analysis and binary logistic regression model have suggested that the important factors from the demographic characteristics that may influence “good” and “not good” classifications in the knowledge dimension were gender and training for GCS usage. Our study indicates that men were more likely to have a good knowledge regarding clinical GCS usage compared with women, and participants who received training on GCS usage had a much higher probability of having good knowledge of its use compared with those who did not receive training. Hence, we believe that the National Program should focus on training to make better use of GCS.

Our study has some limitations. First, participants were from hospitals accredited by the National Program. Their response to the survey was voluntary and not mandatory through the National Program. This study did not investigate the proportion of respondents in the authorized hospital, and the researchers hoped that participants would participate in this survey in a relaxed state. Based on these considerations, our results were likely to be better than those of Chinese studies as a whole. However, this study can still provide a basis for the future work of the National Program to a large extent. In the follow-up study, a cluster sampling survey can be carried out for authorized tertiary hospitals to reflect the current situation more objectively. Second, many of the respondents were nurses (87.1%), most of whom worked in tertiary hospitals (89.2%). China is a developing country with an uneven distribution of medical resources. Generally speaking, medical personnel with high-level knowledge are concentrated in tertiary hospitals. As such, we are concerned about secondary and community hospitals. Hence, the project team faces great work challenges and more doctors are needed to be involved in future research. In addition, a larger study sample is required.

## Conclusions

The knowledge of healthcare professionals regarding GCS clinical usage is not profound, and this represents a barrier for VTE prophylaxis in China. Training for the proper use of GCS in China has not yet met medical staff needs and deserves primary attention from policy makers. The substantial gap in the implementation of the program in the real world may be overcame by gaining the attention of practitioners as well as that of hospital management, which may result in the enhancement of medical education and management regarding GCS clinical usage.

## Supplementary Information

**Additional file 1.**

## Data Availability

The datasets generated and/or analyzed during the current study are not publicly available because they are anonymized, though they are available from the corresponding author on reasonable request.

## Abbreviations

DVT: Deep venous thrombosis
GCS: Graduated compression stockings
PE: Pulmonary embolism
VTE: Venous thromboembolism
