# Supplementary material for: Healthcare professionals’ knowledge, attitudes, and practices regarding graduated compression stockings: a survey of China’s big-data network
Source: BMC Health Serv Res. 2020 Nov 25;20:1078. doi: 10.1186/s12913-020-05933-9 (PMC7690181; doi:10.1186/s12913-020-05933-9)
Supplement: Supplementary file 1 — Additional file 1. [file 12913_2020_5933_MOESM1_ESM.docx]

Part 1: Demographic information

1. Gender:

① Man ② Woman

1. Age:

① < 29 ② 30–39 ③ ≥ 40

1. Highest education attained:

① Secondary ② College ③ Bachelor’s degree ④ Master’s degree ⑤ Doctoral degree

1. Profession:

① Doctor ② Nurse

1. Hospital level:

① Tertiary hospital ② Secondary hospital

1. Service years:

① 1-5 ② 6-10 ③ 11-19 ④ ≥ 20

1. Professional title:

① Junior ② Intermediate ③ Senior

1. Administrative duties:

① None ② Education secretary ③ Head nurse ④ Doctor director

1. Training in the use of GCS:

① Received ② Not received

Part 2: Knowledge (11 items), Attitude (4 items), and Clinical practice patterns (7 items)

*Knowledge (11 items)*

*1.* Mechanism of action:

① very unfamiliar ② unfamiliar ③ generally familiar ④ familiar ⑤ very familiar

*2.* Indications:

① very unfamiliar ② unfamiliar ③ generally familiar ④ familiar ⑤ very familiar

*3.* Contraindications:

① very unfamiliar ② unfamiliar ③ generally familiar ④ familiar ⑤ very familiar

*4.* Size:

① very unfamiliar ② unfamiliar ③ generally familiar ④ familiar ⑤ very familiar

*5.* Pressure level:

① very unfamiliar ② unfamiliar ③ generally familiar ④ familiar ⑤ very familiar

*6.* Length:

① very unfamiliar ② unfamiliar ③ generally familiar ④ familiar ⑤ very familiar

*7.* Timing:

① very unfamiliar ② unfamiliar ③ generally familiar ④ familiar ⑤ very familiar

*8.* Wearing method:

① very unfamiliar ② unfamiliar ③ generally familiar ④ familiar ⑤ very familiar

*9.* Maintenance instructions:

① very unfamiliar ② unfamiliar ③ generally familiar ④ familiar ⑤ very familiar

*10.* Washing method:

① very unfamiliar ② unfamiliar ③ generally familiar ④ familiar ⑤ very familiar

*11.* Service life:

① very unfamiliar ② unfamiliar ③ generally familiar ④ familiar ⑤ very familiar

*Attitude (4 items)*

*1.* The benefits of GCS should be actively communicated to patients and their caregivers:

① strongly disagree ② disagree ③ generally agree ④ agree ⑤ strongly agree

*2.* Medical staff should teach patients and their caregivers the proper use of GCS:

① strongly disagree ② disagree ③ generally agree ④ agree ⑤ strongly agree

*3.* Medical institutions and managers should pay attention to the training of healthcare professionals for the use of GCS:

① strongly disagree ② disagree ③ generally agree ④ agree ⑤ strongly agree

*4.* GCS should be covered by Medicare:

① strongly disagree ② disagree ③ generally agree ④ agree ⑤ strongly agree

*Clinical practice patterns (7 items)*

*1.* I think my guidance for the use of GCS for patients is in place:

① never ② seldom ③ occasionally ④ often ⑤ frequently

*2.* I make sure that the patients in my charge are well informed of the benefits of using GCS:

① never ② seldom ③ occasionally ④ often ⑤ frequently

*3.* I make sure that the patients in my charge are well aware of the importance of the proper use of GCS upon discharge:

① never ② seldom ③ occasionally ④ often ⑤ frequently

*4.* I make sure that the patients in my charge are capable of wearing GCS independently or with the help of the caregivers when they leave the hospital:

① never ② seldom ③ occasionally ④ often ⑤ frequently

*5.* I make sure that the patients under my charge know how to deal with problems (such as skin indentation, blisters or discoloration) associated with the use of GCS, especially at the ankle or protuberance, when they leave the hospital:

① never ② seldom ③ occasionally ④ often ⑤ frequently

*6.* I make sure that at discharge I have made clear to the patients in my charge who should be contacted in case of abnormal use of GCS:

① never ② seldom ③ occasionally ④ often ⑤ frequently

*7.* I make sure that at discharge I have made it clear to the patients in my charge when to stop using GCS:

① never ② seldom ③ occasionally ④ often ⑤ frequently
